# The Role of Stem Cell Factor, Epidermal Growth Factor and Angiopoietin-2 in HBV, HCV, HCC and NAFLD

**DOI:** 10.3390/life12122072

**Published:** 2022-12-09

**Authors:** Leona Radmanić, Snježana Zidovec-Lepej

**Affiliations:** Department of Immunological and Molecular Diagnostics, University Hospital for Infectious Diseases “Dr. Fran Mihaljević”, 10000 Zagreb, Croatia

**Keywords:** growth factors, liver disease, SCF, EGF, Ang-2

## Abstract

Growth factors play a significant role in the immunopathogenesis of liver diseases, especially in liver fibrosis and cirrhosis. They can also play a role in liver regeneration and tissue repair. The regenerative capacity of the liver has been well established. Molecular mechanisms leading to regeneration involve a complex network of diverse molecules. Chronic liver injury leads to the dysregulation of regenerative mechanisms in the liver that, in addition to molecular oncogenesis, lead to uncontrolled cell proliferation and development of hepatocellular carcinoma (HCC). Stem cell factor (SCF), epidermal growth factor (EGF) and Angiopietin-2 (Ang-2) have been shown to be extremely important in the pathogenesis of liver diseases, and given their role in hepatitis B (HBV) or C virus (HCV), HCC and nonalcoholic fatty liver disease (NAFLD), they seem to be potential targets for future research into antifibrotic drugs. The role of SCF receptor c-kit in the liver is debatable, as it has impact on both liver regeneration and liver disease. EGF is a potential indicator of the survival of patients with HCC and can be a biomarker and therapeutic target structure in HCC. Further research is needed to investigate the potential role of Ang-2 for NAFLD associated with liver damage as a non-invasive circulating biomarker.

## 1. Introduction

The microenvironment in the liver that provides a biological background for the development of fibrosis includes several components that are interconnected: stromal cells including immune system cells, fibroblasts, adipocytes and endothelial cells, extracellular matrix (ECM) and biological response modifiers (growth factors, cytokines, matrix metalloproteinases). Growth factors play a significant role in the immunopathogenesis of liver diseases, especially in liver fibrosis and cirrhosis. They can also play a role in liver regeneration and tissue repair and exhibit profibrotic as well as antifibrotic effects [1]. Growth factors with profibrotic effects are angiopoietin 2 (Ang-2), epidermal growth factor (EGF), erythropoietin (EPO), fibroblast growth factor (FGF), granulocyte colony stimulating factor (G-CSF), granulocyte-macrophage colony-stimulating factor (GM-CSF), hepatocyte growth factor (HGF), macrophage colony-stimulating factor (M-CSF), transforming growth factor alpha (TGF-α), transforming growth factor beta (TGF-β), vascular endothelial growth factor (VEGF). A growth factor with antifibrotic effects is stem cell factor (SCF), while platelet-derived growth factor (PDGF) has both profibrotic and antifibrotic properties. The regenerative capacity of the liver has been well established. Molecular mechanisms leading to regeneration involve a complex network of diverse molecules. Chronic liver injury leads to the dysregulation of regenerative mechanisms in the liver that, in addition to molecular oncogenesis, lead to uncontrolled cell proliferation and development of hepatocellular carcinoma (HCC) [2]. HCC most often occurs as a complication of cirrhosis. Therefore, HCC was ranked sixth for cancer incidence and fourth for cancer-related mortality in 2015 [3]. Support in public screening programs, treatment with direct-acting antiviral drugs for hepatitis C infection and routine vaccination against hepatitis B virus (HBV) are extremely important in order to decrease the incidence rate of HCC due to viral hepatitis. However, the prevalence of obesity and metabolic syndrome are increasing, as is the incidence of HCC associated with nonalcoholic fatty liver disease (NAFLD). The highest incidence rates of HCC are in Asia and Africa. Multiethnic countries record significant differences in HCC incidence and mortality [4]. In adults and children, the most common cause of chronic liver disease worldwide is NAFLD. NAFLD includes nonalcoholic fatty liver (NAFL) and nonalcoholic steatohepatitis (NASH), with the latter including steatosis and fibrosis. Nevertheless, the possibility of inflammation reversal is much higher than that of fibrosis reversal. The most important factor is timely recognition and diagnosis of inflammation, which are key to reducing liver disease. It is crucial to distinguish patients with NAFL from those with fibrosis and NASH [5,6]. Fibrosis is less common in children than in adults, and, therefore, the identification of circulating markers is important in order to find patients with NASH who have a higher chance of advanced stage fibrosis. The general prevalence of NAFLD is at 10% in children and at 17% in teenagers. It is an even bigger problem in children and adolescents with obesity, where it amounts to 40–70%. Therefore, NAFLD is now considered to be one of the most common comorbidities associated with obesity mortality [7]. NAFLD is a complex disease that is associated with type 2 diabetes, insulin resistance and other metabolic abnormalities associated with the induction of oxidative stress, hepatic lipotoxicity and systemic and inflammatory liver response [8,9].

In this review, we focus on three growth factors, SCF, EGF and Ang-2, respectively. These have been shown to be extremely important in liver pathogenesis and, considering their role in hepatitis B or C virus (HCV), HCC and NAFLD, they are potential targets for future research into antifibrotic drugs.

## 2. Stem Cell Factor (SCF)

**Figure 1:**
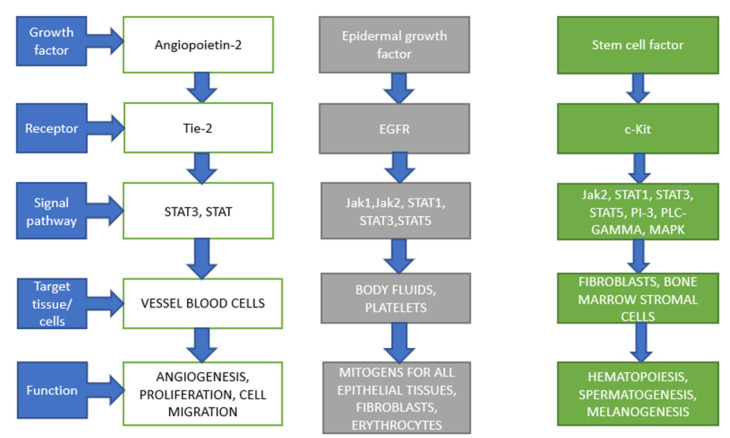
Selected growth factors and their functions.

Stem cell factor, also known as C-kit ligand, steel factor or mast cell growth factor, is a hematopoietic growth factor that promotes survival, proliferation, differentiation and migration of myeloid, erythroid, megakaryocytic, lymphoid, germ cell and melanocyte progenitor cells [10]. It is a crucial regulator of the biology of stem cells involved in hematopoiesis, gametogenesis (oogenesis, folliculogenesis and spermatogenesis) and melanogenesis (proliferation, survival and migration of melanocytes from the neural crest to the dermis) (Figure 1) [11]. As a principal growth factor for mast cells, it promotes their growth, chemotaxis, adhesion and degranulation in vitro [12]. SCF is a four-helical bundle protein that consists of 189 amino acids in a non-covalent homodimer form that is mainly synthesized by endothelial cells, fibroblasts and bone marrow stromal cells. This growth factor is expressed in three forms: a transmembrane form, a soluble 25 kDa protein generated by proteolytic cleavage of the membrane form and a shorter transmembrane form (tmSCF) that lacks the cleavable domain. In addition to structural differences, various isoforms express different amplitudes of biological effects, with tmSCF inducing longer activation of the receptor and longer proliferation of CD34+ hematopoietic cells, in comparison with the soluble SCF [13]. The receptor for SCF is a molecule C-kit (CD117) that is expressed in hematopoietic stem cells, mast cells, germ cells and melanocytes. The initial high-level expression of C-kit during hematopoietic differentiation is gradually decreased and maintained on mast cells, NK-cells and dendritic cells, showing that SCF/C-kit signaling also plays an important role in immune responses [11]. Binding of SCF to C-kit initiates receptor dimerization and autophosphorylation of tyrosine residues in the cytoplasmic domain of the receptor that activates several intracellular signaling cascades, including Janus kinase/signal transducer and the activator of transcription (JAK/STAT), Src kinase, mitogen-activated protein kinase/EC signal-regulated kinase (MAPK/ERK), phosphatidylinositol 3-kinase/protein kinase B (PI3K/AKT) and C–γ (PLC–γ) pathways [14]. In vitro, a combination between a Th2 cytokine IL-4 and SCF enhances the antigen-induced release of mediators from human mast cells, showing an intracellular crosstalk between signaling pathways induced by the two biological response modifiers [15]. Triggering of the Ras/MAPK pathway plays an important role in the melanogenesis and melanosome transfer of melanocytes. Loss-of-function mutations of the C-kit gene are associated with the development of piebaldism, an autosomal dominant genetic disorder of pigmentation in humans. Investigations into SCF as therapeutics have been limited due to concerns about mast cell activation and possible in vivo consequences in humans. However, as recently shown by Takematsu et al. (2022), tmSCF can enhance revascularization in ischemia without massive activation of mast cells by using novel drug delivery systems, including lipid nanodiscs or proteoliposomes [13].

## 3. Epidermal Growth Factor (EGF)

Epidermal growth factor is a member of the EGF family that includes a variety of well-characterized biological response modifiers, including transforming growth factor-α (TGF-α), heparin-binding EGF-like growth factor (HB-EGF), amphiregulin, epiregulin, betacellulin, neuregulins (heregulins, HRGs), epigen and other factors [16]. It is one of the most important factors which regulates cellular proliferation, growth, survival, migration and differentiation. EGF binds to subunits of receptor EGFR (ErbB1, HER1) (Figure 1), a member of the membrane intrinsic tyrosine kinase receptors ErbB, that also includes ErbB2 (HER2), ErbB3 (HER3), and ErbB4 (HER4) [17]. Furthermore, EGF is produced in large amounts by the salivary glands and pancreas [18], which leads to the maintenance of mucosal integrity in the oral cavity, esophagus, stomach and intestines. EGF deficiency, as a result of saliva deficiency, impairs the regeneration of mucosa and increases its damage [19], while EGF administration protects and accelerates the regeneration of gastrointestinal organs. This effect has been found in the oral cavity [20], stomach [21], duodenum [22], liver [23] and pancreas [24], among others. Following EGF/EGFR binding, the complex forms homodimers or heterodimers with other ErbB receptors (ErbB2, ErbB3, ErbB4), or other biologically-related molecules, including PDGFR-β, receptor for hepatocyte growth factor (HGFR/c-MET) and receptor for insulin-like growth factor (IGF)-I [16]. EGFR is expressed in a variety of human cells, including fibroblasts, endothelial cells, keratinocytes, skeletal muscle cells, hepatocytes and cultured 3T3-L1 adipocytes. EGF initiates a variety of intracellular signaling pathways that interact with signaling cascades of other growth factors, cytokines and biological response modifiers, including the RAS–RAF–MEK–MAPK pathway, PI3K–Akt pathway and JAK–STAT signaling cascade [17]. Molecular signaling of this complex signaling network is associated with a variety of EGF biological effects on target cells, including the following: promotion of cellular proliferation (by suppressing cellular senescence), migration of cells (epithelial cells and fibroblasts) to the sites of tissue injury, directly or indirectly, by modifying the composition of the extracellular matrix, promotion of wound healing by inducing activation and proliferation of myofibroblasts, stimulation of re-epithelialization, by promoting keratinocyte motility, de novo angiogenesis, maturation of adipocytes, regulation of thymocyte differentiation, mammary gland lactogenesis and cellular metabolism, including lipogenesis and biosynthesis of glucose and amino acids [16,25,26]. Due to the exceptional complexity and diversity of its biological effects, aberrant EGF is considered to be one of the key molecular mediators of neoplastic transformation of cells. Aberrant EGFR expression and activation play an important role in the development and progression of various cancers. Treatments based on EGFR-targeting kinase inhibitors and monoclonal antibodies are extensively used in selected human cancers (including lung cancer, head and neck cancer and colorectal cancer) [17,27,28].

## 4. Angiopietin-2

Angiopoietins are members of the functionally-related growth factors that play an important role in embryonic and postnatal physiological and pathological angiogenesis, and the regulation of microvascular permeability and inflammation, which includes four members (ANGPT1-4), as well as 8 angiopoietin-related proteins (ANGPTL1-8) [29]. Biological activity of angiopoietins is mediated by Tie-1 and Tie-2 tyrosine kinase receptor signaling pathways (Figure 1). Tie-2 is the main receptor for ANGPT1-4, while Tie-1 is considered an orphan receptor that forms a heterodimer with Tie-2 and is thought to enhance and modulate this signal transduction pathway, particularly in the context of vascular development and maturation [29,30]. Binding of Ang-1 to Tie-2 induces an intracellular signaling cascade mediated by Akt that, subsequently, regulates diverse biological effects of this mediator. Tie-2 is highly expressed in vascular endothelial cells and gene deficiency of Tie-2 leads to abnormal vascular development and embryonic lethality. Ang-1 is a pro-angiogenic factor, synthesized mainly by vascular support cells, specialized pericytes in the kidney and hepatic stellate cells, that is critical for vessel maturation and enlargement, promotion of vascular stability and integrity, and the induction of endothelial cell migration, adhesion and survival [30]. The role of Ang-2 in human physiology is more complex and its biological effect depends on its interaction with other growth factors. Ang-2 promotes cell death and vascular regression by disruption of the connection between the endothelium and perivascular cells, but, in the presence of VEGF, it can promote neo-vascularization. Ang-2 is an important regulator of adipocyte physiology and inflammatory reactions, and its aberrant expression is an important step in the pathogenesis of malignant diseases [31]. Mutations in the genes coding for angiopoietins and angiopoietin-related proteins have been implicated in the pathogenesis of several diseases in humans, such as congenital vascular malformations of the skin and mucosa port-wine stains (angiopoietin-2 in the presence of somatic GNAQ (R183Q) mutations) and familiar combined hypolipidemia (ANGPTL3 deficiency) [32,33]. Due to the important role of angiopoietins and their receptors in angiogenesis, their signaling pathways are an important therapeutic target in experimental clinical oncology. In addition, intravitreal application of faricimab, a bispecific antibody that targets VEGF-A and Ang-2, has been recently approved for the treatment of neovascular age-related macular degeneration or diabetic macular edema, showing a promising therapeutic potential for this group of growth factors [34].

## 5. Molecular and Cellular Mechanisms in the Immunopathogenesis of Liver Fibrosis

The liver is the central organ of the human body responsible for homeostasis, and the only internal organ that shows the ability to regenerate [35]. Liver cells are divided into two groups, parenchymal and non-parenchymal cells. Liver parenchymal cells, i.e., hepatocytes, contain about 60% of the total number of liver cells and account for about 80% of the total cellular volume of the liver [35]. The most important biological functions of hepatocytes are biotransformation and detoxification of xenobiotics, lipid and carbohydrate metabolic cycles and bile synthesis [36,37]. Hepatic sinusoids are surrounded by fenestrated endothelial cells containing cells of the immune system which are non-parenchymal liver cells. Non-parenchymal liver cells contain about 35% of the total number of cells and about 17% of the total cell volume of the liver. This group of cells includes liver sinusoidal endothelial cells (LSEC, about 44%), resident macrophages of the liver or Kupffer cells (about 33%), hepatic stellate cells (HSC, 10–25%) and hepatic NK-cells (5%) [35]. LSEC cells play a particularly significant role in physiological processes in the liver, as well as in innate and specific local immunity, where they act as antigen-presenter cells, stimulate diapedesis of leukocytes, through adhesion molecules and chemokines, and play an important role in the processes of fibrinogenesis and oncogenesis in the liver [38]. Fibrinogenesis is a complex physiological process of healing damaged liver tissue, during which the components of the extracellular matrix (ECM) are secreted into the perisinusoidal spaces (spaces of Disse) to isolate the damaged part of the tissue, by creating a capsule with infiltration cells of the immune system, and to start the tissue recovery processes. Molecular mechanisms of the process of fibrinogenesis have been investigated in detail and include intercellular interactions between hepatocytes, non-liver parenchymal cells and cells of the immune system, as well as the activation and modulation of signaling biological response modulator pathways. In a healthy liver, the ECM contains glycoproteins, i.e., fibronectin, laminin, non-fibrogenic collagen type IV and proteoglycans, such as heparan sulfate. ECM components are organized into a petal-like matrix which enables the maintenance of the structure of the liver parenchyma and intercellular communication. Activation fibrinogenesis after hepatocyte damage causes significant changes in the kinetics of component synthesis of ECM from a special cell population of myofibroblasts. Long-term activation of fibrinogenesis pathways in the liver during chronic hepatitis C (CHC) causes significant changes in the composition of ECM, i.e., the replacement of non-fibrogenic collagen type IV with fibrogenic collagen types I and II, with increased secretion of fibronectin, hyaluronic acid and α-actin of smooth muscle cells [35]. Increased synthesis and incorporation of type I collagen into the ECM ultimately causes physiologic liver changes characteristic of fibrosis. Enzymes from the family of matrix metalloproteinases (MMPs) and collagenase enable enzymatic degradation of ECM components in healthy liver. In the fibrotic liver, myofibroblasts synthesize enzymes from the group of tissue inhibitors metalloproteinase (TIMP1) that inhibit activity of MMP enzymes and prevent enzymatic degradation of ECM. During fibrinogenesis, morphological changes of liver cells occur, of which the defenestration of endothelial cells and the loss of microvilli on the basement membrane of hepatocytes are particularly significant, and these changes cause difficulties in exchange of nutrients, metabolites and signaling molecules operating between hepatocytes and blood. The deposition of type I collagen around the liver lobes, and within the sinusoidal spaces in the liver, changes structural organization of hepatocytes in the liver, prevents the exchange of nutrients and signal molecules from the blood through sinusoidal spaces to hepatocytes and prevents their biological functions. The accumulation of collagen increases the mechanical hardness of the ECM which causes hepatic vasoconstriction blood vessels (especially hepatic portal veins) and leads to portal hypertension, which is associated with clinically significant complications of liver disease, including decompensated cirrhosis [35]. In a healthy liver, HSCs have a quiescent phenotype, and their most significant biological function is storage of vitamin A. A key step in the pathogenesis of liver fibrosis is the activation of HSCs through soluble inflammatory mediators and their differentiation into myofibroblasts, which are the most important cell source of ECM components [39,40,41]. The results of experimental in vivo models show that HSCs are the most significant profibrogenic cells in the liver from which 82–96% of myofibroblasts differentiate [42]. The expression of the α-SMA molecule is used as a molecular marker of HSC activation, but more recent research, using the RNA sequencing method of individual cells (single cell RNA-sequencing, scRNA-seq), showed the existence of phenotypically and functionally different subpopulations of resting HSC and activated myofibroblasts, whose contribution to profibrotic changes in the liver is not yet known [43]. Activation of hepatic stellate cells is the major cellular source of matrix protein-secreting myofibroblasts, the major cause of liver fibrogenesis. Products which can directly, or indirectly, induce stellate cell activation are paracrine signals from injured epithelial cells, the fibrotic tissue microenvironment, immune and systemic metabolic dysregulation, enteric dysbiosis and hepatitis viral products. Dysregulated intracellular signaling, epigenetic changes and cellular stress response represent candidate targets to deactivate stellate cells by inducing reversion to an inactivated state, cellular senescence, apoptosis, and/or clearance by immune cells [44]. In addition to activated HSCs, other liver cell populations can also differentiate into myofibroblasts, such as endogenous portal fibroblasts, fibrocytes, vascular smooth muscle cells, fibrocytes that differentiate from bone marrow cells, biliary epithelial cells, resident liver progenitor cells, sinusoidal endothelial cells, mesothelial cells and perivascular mesenchymal cells [45]. The results of recent research show that, during fibrinogenesis, the differentiation of myofibroblasts from hepatocytes occurs in the process of transition from epithelial to mesenchymal phenotypes, whereby hepatocytes become the cell source of ECM components [46]. Histologically, the degrees of fibrosis are determined according to METAVIR systematization in 5 grades, from F0 to F4. A healthy liver contains collagen connective tissue in portal spaces, around blood vessels and bile ducts and under Glisson’s capsule. Chronic liver diseases lead to the deposition of connective tissue, damage and necrosis of the liver cells, during which the disease progresses to higher degrees of fibrosis and cirrhosis [39].

### Liver Regeneration

Functional and morphological recovery of the fibrotic liver, which was described after the cure of CHC, at the cellular level, can be associated with apoptosis of myofibroblasts in the liver or their differentiation into a resting phenotype. Besides the reduction of the myofibroblast population, increased synthesis of enzymes of the MMP family in macrophages also has a significant role in recovery. Regeneration of liver tissue is mediated by progenitor cells that are generated by the transdifferentiation of biliary epithelial cells. The main biological function of resident liver progenitor cells is the differentiation into hepatocytes and cholangiocytes that enable regeneration of tissues of the damaged liver. In vitro research also showed that hepatocytes can differentiate into cells similar to progenitor liver cells, which gave rise to the hypothesis that hepatocytes are facultative progenitor liver cells [47]. Molecular mechanisms of proliferation or stimulation of hepatocytes during the process of liver regeneration have not been fully investigated, but it is assumed that they enable signaling pathways activated by EGFR and MET receptors [48]. Interaction of different parenchymal and non-parenchymal liver cells and cells of the immune system that migrate to the liver is mediated by the responses of biological modulators. The most important modulators are cytokines, chemokines and growth factors, which are investigated as possible targets of innovative antifibrotic drugs [39].

## 6. SCF in Liver Disease

**Figure 2:**
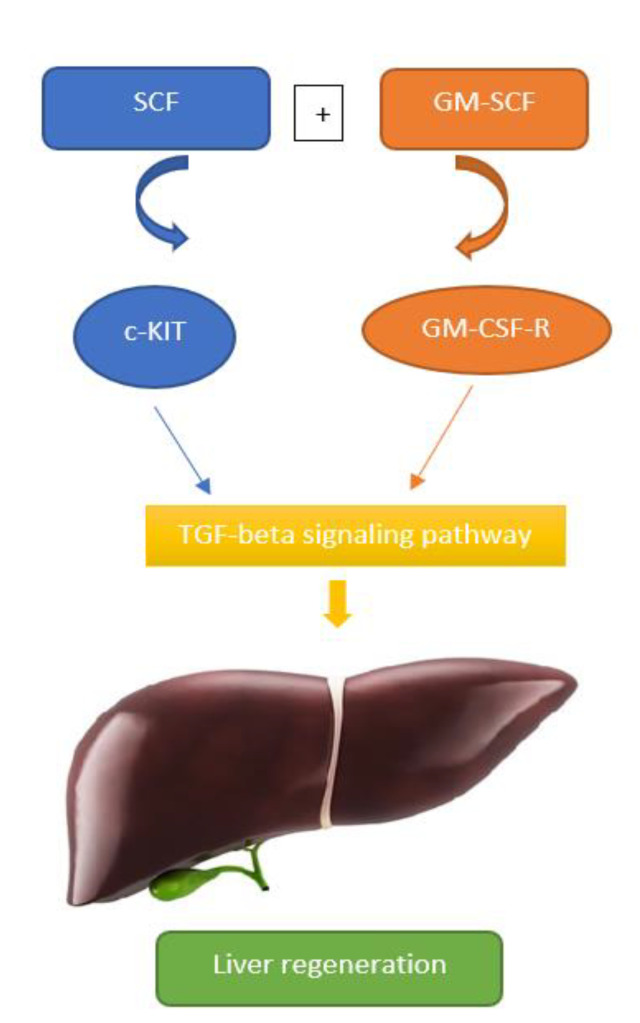
Stem cell factor (SCF) and granulocyte-macrophage colony-stimulating factor (GM-CSF) depends on the transforming growth factor beta (TGF-β) signaling pathway that contributes to liver regeneration.

SCF achieves its biological effect by binding to the c-Kit receptor and regulates the differentiation of CD34+ stem cells. Moreover, SCF shows antifibrotic effects in patients with CHC. The important biological activities of SCF are stimulation of the synthesis of pro-inflammatory cytokines, chemokines and histamine in mast cells located in the skin and mucous membrane epithelium. The biological activity of SCF is associated with the Th2 cytokine IL-4, which prevents the differentiation of cultured fetal liver mast cells stimulated with this growth factor, by reducing the expression of the c-Kit receptor [49]. Stem cell factor plays a significant role in liver regeneration. In combination with GM–CSF, SCF synergistically stimulates the proliferation and differentiation of cholangiocytes, plays an important role in stimulating the proliferation and migration of mesenchymal cells and regulates the activity of enzymes that play an important role in modulating the matrix, such as tissue inhibitor of metalloproteinase-3 (TIMP-3), matrix metalloproteinase-2 (MMP-2) and matrix metalloproteinase-9 (MMP-9). Furthermore, SCF plays an important role in the regeneration and remodeling of the liver [50]. Gaca et al. showed that HSC activated in vitro produced SCF and that these cells may play an important role in recruiting mast cells in a response to the liver during injury and fibrosis [51]. Meadows et al. demonstrated that cholangiocytes secrete SCF, which is expressed on mast cell infiltration as a chemoattractant, contributing to hepatic fibrosis [52]. Radmanic et al. characterized SCF as a potential biomarker in CHC of direct acting antivirals (DAA) treatment [53]. In 2012, a group of scientists from Japan, Korea and China hypothesized that SCF is one of the most important factors in liver tissue remodeling, along with EPO, G-CSF and GM–CSF [50]. These cytokines act on stem cells leading to cell differentiation. Growth factors SCF and GM–CSF are important regulators of liver tissue remodeling and play a synergistic role in liver regeneration. The synergistic effects are important because hematopoietic stem cells and early progenitor cells require the combined action of SCF and GM–CSF for self-renewal and differentiation. The synergy of SCF and GM–CSF depends on the TGF–β signaling pathway that contributes to liver regeneration (Figure 2) [50]. Increased expression of SCF in patients with CHC is also associated with increased expression of the Th2 cytokine IL-4, which affects the phenotype of activated fibroblasts, by inducing the appearance of myofibroblasts [50]. Radmanic et al. demonstrated that the SCF level was significantly increased in patients with CHC, compared to healthy individuals, and suggested that SCF could contribute to liver repair in CHC [53]. Stem cell factor is a hematopoietic cytokine which causes proliferation and differentiation of cells by binding to c-kit tyrosine kinase receptor. SCF and c-kit receptor RNA are expressed in the fetus and in early germ cells, including the fetal liver during the embryonic period. Defects in SCF and its signaling pathways lead to impaired hematopoiesis [54]. The SCF/c-kit signaling pathway has an important role in cell proliferation, migration and survival, thus, influencing hematopoiesis, pigmentation, and spermatogenesis. The role of the c-kit in the liver is debatable, as it impacts on both liver regeneration and liver disease. Firstly, liver c-kit+ cells, including parts of hepatocytes, participate in liver tissue repair by regenerating target cells. Simultaneously, c-kit+ mast cells play a crucial role in liver fibrosis. Moreover, c-kit is also a proto-oncogene. Nevertheless, the role of c-kit in the liver is insufficiently researched [10]. Rybtsov et al. demonstrated that SCF is the key factor in the maturation of HSCs [55]. The only visceral organ that has the capacity to regenerate is the liver. About 20% of hepatocytes express c-kit which participates in liver regeneration. The progression of liver disease is associated with pathological angiogenesis which is a precondition for the development of HCC [56]. Moreover, stem cell growth factor-beta (SCGF-β) shows activity on granulocyte/macrophage progenitor cells in combination with GM-CSF and M-CSF. Therefore, obesity-associated inflammation induces insulin resistance (IR), which is crucial to NAFLD or hepatic steatosis (HS) [57]. Tarantino et al. demonstrated that SCGF-β levels were linked to IR and HS severity. Prediction of HOMA values by SCGF-β levels, likely mediated by markers of inflammation, is shedding some light on the mechanisms inducing/worsening IR of male patients with obesity-related NAFLD [58].

## 7. EGF in Liver Disease

**Figure 3:**
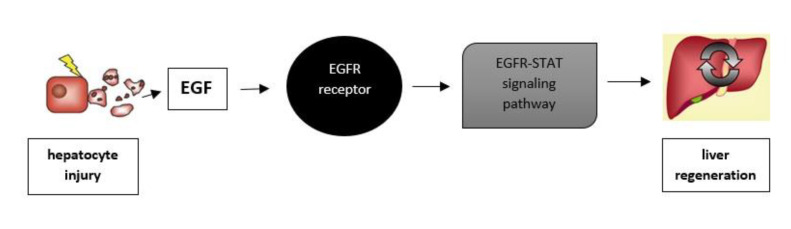
Epidermal growth factor receptor (EGFR) contributes, through the EGFR/signal transducer and activator of transcription proteins (STAT) signaling pathway, to liver regeneration after liver injury.

The growth factor EGF promotes the growth of hepatic stellate cells and development of liver fibrosis [59]. Shehata et al. demonstrated elevated serum EGF concentrations in 30 patients with HCC and CHC compared to 20 patients with CHC without HCC and 20 healthy individuals, indicating a possible role of this growth factor in oncogenesis associated with chronic viral hepatitis [60]. Yang et al. showed that in serum samples using the ELISA method from 110 patients with CHC and HCC, EGF is a potential indicator of the survival of patients with HCC and can be a biomarker and therapeutic target structure in HCC. The associations between serum EGF concentrations and vascular invasion and extrahepatic metastases were statistically significant [61]. In a study conducted in 2013, Shehata et al. found that serum EGF concentrations were significantly higher in patients with HCC compared to patients with CHC and controls (Table 1). The obtained results show that elevated EGF concentration is characteristic for cirrhosis [60]. The growth factor EGF plays an important role in tumorigenesis, including HCC. Furthermore, EGF gene diversity is associated with susceptibility to several types of cancer [62]. Radmanic et al. showed that the concentration of EGF was the highest after the treatment of CHC infection in 56 patients, so they suggest a possible role of EGF in liver tissue repair after treatment with DAA [53]. The liver has a remarkable ability to regenerate in response to the loss of necrotic tissue. EGFR plays a key role in liver regeneration and response to liver injury. High levels of EGFR are expressed by hepatocytes, and deletion of EGFR leads to embryonic death shortly after birth, depending on genetic association with liver disorders. Reduced liver weight results in perinatal deletion of EGFR in hepatocytes. EGFR expression is increased in partial hepatectomy. EGFR also contributes, through the EGFR/STAT signaling pathway, to regeneration after liver injury and protects the liver from alcohol-induced injury (Figure 3). Activation of a chronic stimuli to the hepatic wound-healing response can lead to liver fibrosis [63]. In a recent study, it was shown that EGFR is needed in liver macrophages to induce the transcription of interleukin 6, which is important for inducing hepatocyte proliferation and hepatocellular carcinoma. EGFR activation is associated with a poor prognosis in carcinomas of the biliary tract. Bile acids induce cyclooxygenase-2 expression through EGFR/MAPK signaling, which is known as an inducer tumorigenesis of many types in a human cholangiocarcinoma cell line [17]. El Taghdouini et al. demonstrated the ability of human primary aHSCs to revert in vitro to a transitional state through synergistic action of EGF, FGF2, dietary fatty acids and retinol, and provided the first phenotypic and genomic characterization of human in vitro reverted HSCs [64].

## 8. Angiopoietin-2 in Liver Disease

**Figure 4:**
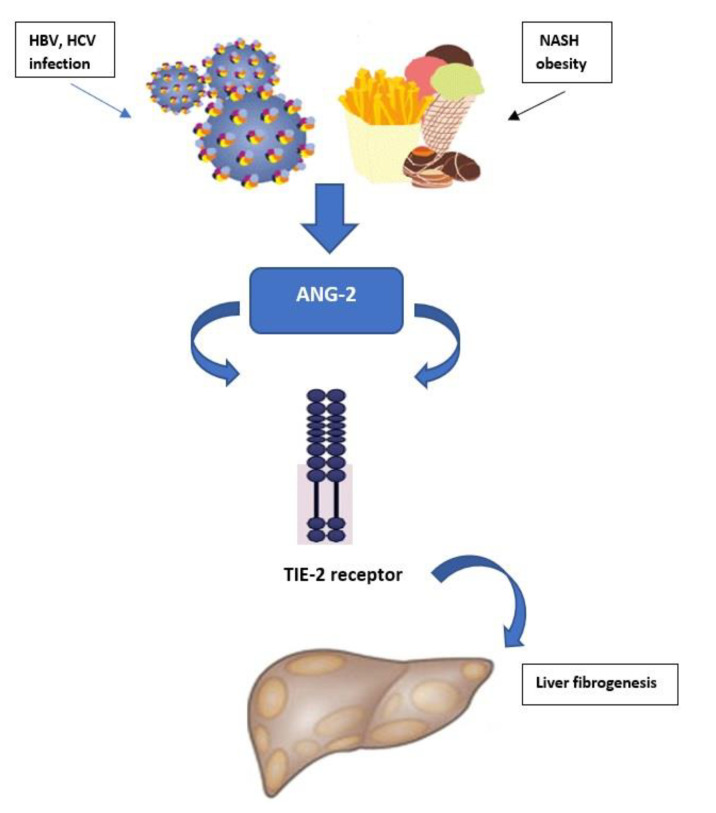
Higher serum of angiopoietin 2 (Ang-2) concentration in patients with hepatitis B virus (HBV) or hepatitis C virus (HCV) and nonalcoholic steatohepatitis (NASH) contributes to liver fibrogenesis.

Angiopoietins 1-4 (Ang 1-4) belong to a family of growth factors that exert their biological effects through receptors Tie-1 and Tie-2 with tyrosine kinase activity. The factor Ang-1 exhibits a strong angiogenic effect by binding to the Tie-2 receptor, while Ang-2 binding to the identical receptor exhibits an antagonistic effect to the effects of Ang-1 [68]. The synthesis of Ang-2 is stimulated by inflammatory mediators, and it is important for the physiology of endothelial cells, whereby it regulates the permeability of the endothelium [69]. Hernández-Bartolomé et al. showed that the ratio of Ang-2 and Ang-1 concentrations is related to the degree of fibrosis in patients with CHC, which indirectly showed that these molecules were important in the pathogenesis of the disease [65]. Hernández-Bartolomé et al. analyzed serum Ang-2 concentrations as a biomarker of CHC cirrhosis. Serum Ang-2 expression was analyzed in samples of 179 CHC patients with and without liver cirrhosis. Serum Ang-2 concentration in patients with CHC was significantly increased in patients with cirrhosis (Table 1) [65]. Radmanic et al. demonstrated that patients with chronic hepatitis C with severe fibrosis stages exhibited higher serum concentration of Ang-2 [53]. This finding was in line with the study from Hernández-Bartolomé et al. which showed that Ang-2 could be important in severe HCC pathogenesis (Figure 4) [65]. Ang-2 plays an important role in hepatic angiogenesis and vascular permeability from parenchymal and non-parenchymal cells in hypoxia, chronic inflammation, fibrosis and liver injury. Ang-2 was overexpressed in a fibrotic liver, which indicated the role of this factor as a potential target for reducing angiogenesis and liver inflammation during fibrogenesis [70,71]. Lefere et al. analyzed the role of Ang-2 in angiogenesis, leukocyte infiltration and inflammation during NASH. The research showed that patients with NASH had elevated levels of Ang-2. compared to patients with steatosis or obese patients with a diagnosis of NAFLD, and that elevated serum levels were correlated with degrees of steatosis, but not with fibrosis. Therefore, they suggested that further research was needed to investigate the potential role of Ang-2 for NAFLD associated with liver damage as a non-invasive circulating biomarker [66]. Manco et al. investigated the role of Ang-2 as a biomarker for pediatric NAFLD-related liver damage. The mean plasma levels of Ang-2 were higher in children with NAFLD than in age-matched controls. Ang-2 was significantly increased in children with NASH, so they suggested that Ang-2 could be a suitable biomarker of NASH in the pediatric population (Figure 4) [67]. Pocino et al. suggested that serum angiogenic markers, especially Ang-1/2, can contribute to the development of quantitative methods for determining the degree of liver disease and monitoring the effect of therapy [56]. Kimura et al. showed that Ang-1 and Ang-2 may be involved in both reconstruction and capillarization of the sinusoids in rat liver after partial resection and necrosis through interaction between HSC and sinusoidal and vascular endothelial cells by means of Tie-2 receptor [72].

## 9. Conclusions

The only visceral organ in the human body with the capacity to regenerate is the liver. Therefore, it is crucial to investigate the potential roles of growth factors in liver fibrosis and cirrhosis as possible targets of innovative antifibrotic drugs. The progression of liver disease is a precondition for the development of HCC. The role of SCF receptor c-kit in the liver is debatable, as it impacts on both liver regeneration and liver disease. Furthermore, the receptor for EGF plays a key role in liver regeneration and response to liver injury. Moreover, research has indicated the role of Ang-2 as a potential target for reducing angiogenesis and liver inflammation during fibrogenesis. Ang-2 could be a suitable biomarker of NASH in the pediatric population. Therefore, further research is required in order to better understand the role of growth factors in liver diseases.

## Figures and Tables

**Table 1:** Review of scientific papers related to angiopoietin-2, epidermal growth factor and stem cell factor in hepatitis B, C, hepatocellular carcinoma and nonalcoholic fatty liver disease.

	Study	Design	Main Outcomes	Conclusions and Implications
**HEPATITIS B/C and/or hepatocellular carcinoma**	Radmanić et al., 2022 [53]	Retrospective study, 56 patients with chronic hepatitis C and 15 controls	Significantly higher levels in hepatitis C patients after reaching SVR when compared to healthy individuals were showm in Ang-2 (medians 2180.4 and 1206.6 pg/mL, *p* = 0.027), EGF (medians 73.4 and 30.8 pg/mL, *p* = 0.037) and SCF (medians 86.5 and 28.6 pg/mL, *p* < 0.001).	Tje results suggested promotion of liver regeneration in chronic hepatitis C patients during direct acting antiviral treatment.
	Shehata et al., 2013 [60]	Retrospective study, 20 healthy volunteers, patients are subdivided into 20 patients with chronic hepatitis C infection and 30 patients with hepatocellular carcinoma	Significantly higher serum levels of EGF in patients with HCC (1476 ± 970 pg/mL), compared to the level in patients with chronic hepatitis C infection (747 ± 296 pg/mL) and control subjects (625 ± 175 pg/mL).	EGF can be used as a sensitive biomarker in the diagnosis, prognosis, metastasis and recurrence of hepatocellular carcinoma patients and in the management of hepatocellular carcinoma.
	Yang et al., 2018 [61]	Retrospective study, 182 cases of hepatocellular carcinoma formalin-fixed and paraffin-embedded tissues and 110 cases of hepatocellular carcinoma/chronic hepatitis C serum samples	The correlations between serum EGF and vascular invasion (32 pg/mL) and extrahepatic metastasis (31.7 pg/mL) were statistically significant (*p* < 0.0001).	EGF is a potential indicator of the survival of patients with hepatocellular carcinoma and can be a biomarker and therapeutic target structure in hepatocellular carcinoma.
	Li et al., 2010 [62]	Retrospective study,186 hepatitis C/hepatitis B patients, 152 cirrhotic patients with hepatitis B and 186 healthy individuals	Mean level of EGF protein (61 A/G polymorphism) in the hepatocellular carcinoma cell lysate with the GG genotype was higher than that with the AA genotype (47 vs. 32 pg/mL).	EGF gene diversity is associated with susceptibility to several types of cancer.
	Hernández-Bartolomé et al., 2016 [65]	Prospective study, 179 cirrhotic and non-cirrhotic chronic hepatitis C patients	Ang-2 was significantly increased as chronic hepatitis C progressed to the end stage of liver disease (6000 pg/mL, *p* < 0.001).	Ang-2/Ang-1 ratio might be useful for monitoring the progression of chronic liver disease and plays an important role as a therapeutic target.
	Pocino et al., 2021 [56]	Non-profit interventional study, 62 patients, out of whom 33 were diagnosed with hepatocellular carcinoma and 29 with liver cirrhosis	A reduction of Ang-2 (0.047 pg/mL) levels and the Ang-2/Ang-1 ratio (0.031 pg/mL) was observed before and after the treatment of patients diagnosed with viral hepatitis with the required antiviral treatment.	Serum angiogenic markers, with emphasis on Ang-1/2, can contribute to the development of quantitative tools for liver disease staging and therapy monitoring.
**NAFLD**	Lefere et al., 2019 [66]	Prospective study, 13 patients with no nonalcoholic fatty liver disease, 41 patients with nonalcoholic fatty liver, 50 patients with nonalcoholic steatohepatitis	Serum Ang-2 levels were increased in patients with histological nonalcoholic steatohepatitis (400.4 pg/mL (279.2–630.2), compared with patients with simple steatosis (249.8 pg/mL (182.4–317-9).	Ang-2 inhibition could be a therapeutic strategy to target pathological angiogenesis in nonalcoholic steatohepatitis.
	Manco et al., 2022 [67]	Observational study, children with diagnosis of nonalcoholic fatty liver disease (N = 76), control group (N = 28) included children negative to steatosis	The mean plasma level of Ang-2 was higher in children with nonalcoholic fatty liver disease than in age-matched controls (Ang-2 155.4 ± 72.5 vs. 7.5 ± 2.3 ng/mL, *p* < 0.001).	Ang-2 could be a suitable biomarker of nonalcoholic steatohepatitis in the pediatric population.

EGF = epidermal growth factor; SCF = stem cell factor, Ang-2 = angiopoietin 2.

## Data Availability

Not applicable.
